# Exploring Cutaneous Melanoma Patients’ Experiences with Follow-Up Radiology: A Qualitative Study

**DOI:** 10.3390/healthcare13080845

**Published:** 2025-04-08

**Authors:** Laura Iacorossi, Simona Molinaro, Francesca Gambalunga, Fabrizio Petrone, Flora Desiderio, Francesca Piacentini, Antonino Guerrisi, Fulvia Elia, Mauro Caterino

**Affiliations:** 1 Department of Life, Health and Health Professions Sciences, Link Campus University, 00165 Rome, Italy; l.iacorossi@unilink.it; 2 Nursing Research Unit IFO, IRCCS Regina Elena National Cancer Institute, Via Elio Chianesi 53, 00144 Rome, Italy; simona.molinaro@ifo.it; 3 Professional Health Care Services Department, University Hospital “Policlinico Umberto I”, 00161 Rome, Italy; 4 Nursing, Technical, Rehabilitation, Assistance and Research Direction, IRCCS Istituti Fisioterapici Ospitalieri, IFO, 00144 Rome, Italy; fabrizio.petrone@ifo.it; 5 Radiology and Diagnostic Imaging Unit, Department of Clinical and Dermatological Research, San Gallicano Dermatological Institute IRCCS, Via Elio Chianesi 53, 00144 Rome, Italy; flora.desiderio@ifo.it (F.D.); francesca.piacentini@ifo.it (F.P.); antonino.guerrisi@ifo.it (A.G.); fulvia.elia@ifo.it (F.E.); mauro.caterino@ifo.it (M.C.)

**Keywords:** cutaneous melanoma, emotional distress, patient experience, diagnostic imaging, psychological adaptation, qualitative research

## Abstract

**Background/Objectives:** Diagnostic examinations that cutaneous melanoma (CM) patients undergo during follow-up can generate various emotional states related to the possibility of recurrence or disease progression. Understanding the emotions and perceptions of patients during the wait for diagnostic exams is crucial for improving care. This study aims to explore the experiences of CM patients awaiting follow-up diagnostic investigations. **Methods:** This is a qualitative descriptive inductive, single-center study, structured according to COREQ criteria. The sample of CM patients was recruited at the Radiology and Diagnostic Imaging Unit of the San Gallicano Dermatological Institute in Rome. Data were collected through semi-structured interviews and analyzed using Framework Analysis, as described by Ritchie and Spencer. **Results:** A total of 30 patients with an average age of 57.4 were recruited. The interviews revealed the following four themes: waiting influences the emotional experience; the double face of family support; strategies of support during the waiting period; and waiting requires attention to the person and the environment. **Conclusions:** This study revealed that waiting for a diagnostic exam for cutaneous melanoma (CM) is often characterized by anxiety and worry. Healthcare professionals need to implement positive coping strategies, balanced family support, and improved communication to enhance patient care and satisfaction in oncology settings. Recognizing and addressing patients’ emotional dynamics is crucial for improving care. Training healthcare staff, providing psychological interventions, and leveraging technological innovation can improve the waiting experience, foster a more welcoming and reassuring environment, and enhance patients’ psychological well-being and satisfaction with care pathways.

## 1. Introduction

Cutaneous melanoma (CM) is one of the most common cancers among young people, ranking as the second most common cancer among men and the third among women under 50 in Italy [1]. According to the latest AIOM estimates, around 12,700 new cases of melanoma were registered in 2022, with an annual increase in incidence of 4.4% in men and 3.1% in women [1]. Incidence rates vary significantly from north to south, with rates up to twice as low in southern regions [1]. Internationally, the incidence of CM is rising, with Northern European countries like Norway and Sweden showing some of the highest rates worldwide, likely linked to fair skin and increased UV exposure [2]. Studies from Germany and other regions confirm similar trends of growth, underlining the global health challenge posed by CM [3]. Over the last 40 years, the incidence of CM has increased sixfold, especially among fair-skinned individuals [2,4].

The early detection of primary CM, recurrences, and locoregional metastases is essential for improving patient outcomes. Evidence suggests that timely follow-up radiological examinations, such as ultrasound, CT scans, and PET/CT, play a key role in monitoring disease progression and reducing recurrence-related mortality [5]. About 10% of patients develop recurrences, 85% of which occur within five years from the excision of the primary tumor [6].

Although most studies have focused on the clinical and therapeutic aspects of CM, very little data exist on the emotional and psychological impact the disease has on patients, particularly before diagnostic investigations, whose results can significantly influence prognosis and therapy options. Emerging evidence highlights that patients experience high levels of anxiety, particularly during follow-up imaging examinations due to uncertainties about disease progression. For instance, the concept of “anxiety” has been coined to describe the distress patients feel while awaiting imaging results [5]. Anxiety tends to peak during critical diagnostic moments, which underscores the urgent need to address this psychological burden [7].

The role of healthcare professionals, particularly nurses, is crucial in alleviating patient suffering and anxiety while awaiting a diagnosis. Nurses, with their expertise and empathy, can provide significant psychological support by listening to the concerns of patients, explaining the diagnostic process, and offering comfort when needed [8,9].

Such support not only reduces anxiety but also fosters resilience and enhances overall well-being during care processes [7].

Despite the importance of this topic, in-depth data on the perceptions and emotions of patients with CM awaiting follow-up diagnostic investigations are lacking. This study aims to fill this gap by conducting a qualitative exploration of patients’ emotional experiences during follow-up imaging examinations. These insights could inform the development of targeted care strategies that integrate both clinical and psychological support, ultimately improving patient outcomes and quality of life.

## 2. Materials and Methods

### 2.1. Study Design

This is a descriptive, inductive, single-center qualitative study [10], structured according to established Consolidated Criteria for Reporting Qualitative Research (COREQ) [11,12] (Supplementary File S1).

### 2.2. Sample and Setting

Purposive sampling [13] was performed to include patients with cutaneous melanoma who were referred to the UOSD Radiology and Diagnostic Imaging unit at the ISG-IFO of Rome, regardless of the stage of the disease. Inclusion criteria were being over 18 years of age, having a histologically confirmed melanoma diagnosis, being scheduled for a radiological investigation (Ultrasound, CT, PET, MRI), and agreeing to participate in the study (written informed consent). Patients with cognitive impairments or pathological conditions that could hinder active participation in the study were excluded. To identify these patients, medical records were reviewed, and consultations with attending physicians were conducted to ensure potential participants met the inclusion criteria. The sampling was proactive to take advantage of personal experiences of the phenomenon being studied [14]. This approach allows for a comprehensive understanding of the subject of the study, thanks to the participants’ availability, willingness to engage, and ability to express their experiences reflectively and articulately [15]. The sample size was determined based on the principle of data saturation. Data saturation, as proposed by Guest et al. [16], emphasizes the importance of reaching a point where no new information or significant themes emerge from the collected data.

### 2.3. Data Collection

Semi-structured interviews guided by key questions (Table 1) were conducted to explore the phenomenon under investigation. Researchers encouraged participants to share their experiences spontaneously, concluding the interview with a final question and asking them for suggestions. Interviews took place in a dedicated room, ensuring a disruption-free environment that provided comfort and confidentiality to the participants.

The interview questions were developed through an iterative process that included a literature review, expert consultation, and pilot testing. The literature review helped identify knowledge gaps and inform the formulation of the questions. Previous studies highlighted the importance of understanding patients’ emotions during the waiting period for diagnostic exams [17,18]. The interview questions were then reviewed by a panel of experts in oncology and qualitative research. The experts provided feedback on the relevance, comprehensiveness, and appropriateness of the questions, ensuring they were aligned with the research objectives and capable of eliciting meaningful responses [19].

A pilot test was conducted with six participants to evaluate the clarity and effectiveness of the questions [20]. The pilot interviews helped identify any issues and make necessary adjustments to the interview guide [20]. This process improved the quality and credibility of the data collected. During the interviews, the interviewer maintained a degree of flexibility, exploring emerging topics and asking follow-up questions based on participants’ responses. This approach allowed for the collection of rich and in-depth qualitative data [21].

The interviews were conducted by two nurses experienced in qualitative research, who managed the patient recruitment, explained the objectives of the study, and collected the data. Patients were invited to carefully read and sign the informed consent form to ensure an informed decision regarding their participation. Data collection continued until no new information emerged (data saturation).

Before the interview, participants completed a demographic data form (age, gender, education level, marital status, occupation, family structure, number of children) and clinical information (disease, previous oncological therapies, current treatment, type, and reason for the investigation). Interviews were conducted individually, audio-recorded, and later transcribed verbatim using the “smooth verbatim transcription” technique.

### 2.4. Data Analysis

The data were analyzed using the Framework Analysis approach [22]. This method allows the researcher to explore the data in-depth while maintaining effective and transparent control, improving the rigor of the analytical process and the credibility of the results.

Framework Analysis, as described by Ritchie and Spencer [22], is an analytical process consisting of five distinct, highly interconnected stages: familiarization, identifying a thematic framework, indexing, charting, and mapping and interpretation [23]. Table 2 illustrates an example of the coding process applied during the thematic framework stage, showcasing how codes evolved into categories and themes.

Each researcher independently reviewed the interview transcripts and audio recordings, noting key ideas and recurring patterns (familiarization phase). Using these insights, an initial thematic framework was developed to guide subsequent coding and indexing. Data were systematically coded to reflect the identified themes, as shown in Table 2, which includes examples such as “anxiety” and “fear of recurrence.” Summaries and charts were created to organize data under main themes and categories, enabling a deeper interpretation of relationships within the dataset (mapping and interpretation phase).

To ensure consistency and rigor, three researchers conducted the coding and thematic development independently, and all outputs were verified collaboratively. The use of NVivo 15 software facilitated the management and organization of qualitative data but did not replace the interpretative work performed by the researchers. Instead, it provided tools for efficient indexing, searching, and mapping of the data.

### 2.5. Reliability and Validity

To ensure the reliability and validity of the results, various strategies were implemented following the paradigm proposed by Lincoln and Guba [24,25]. Regular meetings among the research team facilitated open discussions, allowing for the identification and mitigation of potential personal biases, contributing to the study’s credibility. Every stage of the research was documented in detail to enable other scholars to replicate the investigation, ensuring reliability. Internal consistency was maintained by sharing the results among researchers using triangulation. The cross-review technique was adopted, where two less-involved researchers analyzed the process by comparing the themes and subthemes that emerged with the original texts. To ensure transferability, clear details about the participants will be provided, allowing readers to apply the results and conclusions to other contexts.

### 2.6. Ethical Considerations

Before the start of the study, approval was obtained from the Lazio Area 5 Territorial Ethics Committee—Experimental Protocol Registry No. 40/ISG/23 on 20/09/2023. Patients who met the inclusion criteria were informed about the objectives of the study and the data collection methods. They were then invited to carefully read the informed consent form to ensure an informed decision was made regarding participation. After written informed consent, data were collected, processed, and stored anonymously to ensure the security and confidentiality of the information of the population involved.

## 3. Results

Thirty patients were interviewed, and the average length of the interview was 24 min. The interviewed sample consisted mostly of men (*n* = 20; 66.67%), with an average age of 57.4 years, married (*n* = 22; 73.33%), and holding a degree (*n* = 17; 56.67%), with employment (*n* = 24; 80.00%). The majority of the sample lived with three to four family members (*n* = 13; 43.33%) and had three or more children (*n* = 13; 43.33%). All patients had received prior oncological therapies, and most were currently undergoing treatment (*n* = 28; 93.33%). The patients we interviewed were awaiting CT scans (*n* = 15; 50.00%), ultrasounds (*n* = 10; 33.33%), and MRIs (*n* = 5; 16.67%) (Table 3).

From the qualitative analysis, four themes emerged:

1. Waiting Affects the Emotional Experience

The waiting period for diagnostic exams represents an emotionally charged experience for patients, marked by a mix of anxiety, fear, and, in some cases, resilience. This time is perceived and experienced differently by each individual, influenced by factors such as personality, past experiences, and the quality of support received from healthcare professionals. Some patients experience the waiting period negatively, perceiving it as excessively long and stressful:

*“I feel awful because the wait is long… many thoughts are running through my mind…”*

(I18)

*“… I’m still waiting for them to call me for the CT scan”*

(I22)

*“… the waiting time is long… the wait has always been devastating”*

(I18)

*“The waiting times… entering and finding out you have to wait another half hour is unbearable. That half hour feels endless.”*

(I2)

Others feel additional stress due to preparation requirements or uncertainty regarding the results:

*“…since it’s an ultrasound where I have to drink, it stresses me a little because I’m not sure if my bladder will be full or if I’ll be able to hold it.”*

(I17)

*“… the wait creates anxiety… will I be able to do the bladder ultrasound?”*

(I23)

*“If something negative comes up, of course, it would lead to a spiral of worries.”*

(I6)

*“If there are significant changes in the report, I would be worried…”*

(I29)

*“… you don’t know if there is something wrong or not, and this creates a state of fear, a constant sense of suspense… there’s no one to tell you everything is fine.”*

(I24)

The predominant negative emotions stem from fear of potential disease progression or recurrence:

*“The fear of recurrence…”*

(I10)

*“… So I’ll have to remove the tumor, I’ll have to go through the whole process again, and so some fear comes in…”*

(I15)

*“… fear of what lies ahead, knowing that these melanomas can cause metastases…”*

(I19)

However, not all patients view the wait negatively. Some adopt a positive outlook or rational approach, which helps them manage their emotions more effectively:

*“What helps is that I’m very positive… I try to face things with a certain philosophy, always thinking of the best…”*

(I7)

*“… I always look on the bright side…”*

(I8)

*“And I’m optimistic, I want to stay calm…”*

(I9)

*“The first few years were more complicated… but now, since 2014, I’ve gotten pretty calm, so I face it with peace of mind.”*

(I6)

*“It also depends a bit on the character of the person; I tend to be rational…”*

(I29)

*“… I’m quite calm, rational by nature…”*

(I15)

Another key factor in reducing anxiety is the perceived competence and kindness of healthcare staff:

*“… the staff I found at IFO is not only competent but much more… they are extremely professional and experienced, which makes you feel calm.”*

(I5)

*“… there is professionalism from everyone…”*

(I12)

*“… when I dealt with the specialists, I calmed down… a competent person can reassure you because they’ve seen thousands of cases like mine…”*

(I19)

*“… the way they handle patients with kindness helps… it helps you relax and face the visit differently.”*

(I17)

*“… kindness… being greeted by a caring and kind person is what we need, it makes you feel relieved…”*

(I27)

*“… I also found kindness; they take care of people… I found reliable people, that’s it”*

(I19)

Also, being provided with the right information makes the wait more positive:

*“… we’re followed so well that we’re calm, always informed about what’s happening and updated on every little detail… information brings peace of mind.”*

(I14)

*“I was reassured, they explained what would happen, what the next steps would be, so I know what I’ve had, what it entails, and what the risks are…”*

(I22)

Finally, trust in scientific progress and advancements in medicine provides reassurance:

*“… there are treatments; we’re in 2024, and fortunately, medicine is progressing, and that helps me overcome fears…”*

(I18)

*“… I believe in science and doctors… I feel calm when I come here.”*

(I5)

*“… and the positive thing is that research is moving forward; I follow the Facebook page and see that a lot of research is being done, which is reassuring…”*

(I10)

The emotional impact of waiting for diagnostic tests is diverse, ranging from intense distress and uncertainty to resilience and optimism. The findings underscore the need for tailored approaches to address patient anxiety, enhance coping mechanisms, and ensure supportive healthcare environments. These elements are key to improving patients’ overall experiences and emotional well-being during the waiting period.

2. The Two Faces of Family Support

Family or friend support plays a dual role in shaping patients’ emotional experiences during the waiting period. On one hand, many patients find reassurance and peace of mind in the presence of loved ones. The emotional stability provided by family members and friends helps them face the challenges of waiting with a sense of calm and resilience:

*“This gives me peace of mind: seeing my children doing well, calm, and then I’m calm too.”*

(I8)

*“… I’m happy like this, with my wife, my daughter, with my family and my daughter’s family, two families, they’re doing well, and so are we.”*

(I20)

*“… I have the fundamental support of my daughter…”*

(I26)

*“I’m very sociable, I have friends, I never lose heart, that helps me, this awareness helps me in society…”.*

(I9)

Being without such support shapes patients’ emotional experiences during the waiting period. On one hand, many patients find reassurance and peace of mind in the presence of loved ones. The emotional stability provided by family members and friends helps them face the challenges of waiting with a sense of calm and resilience:

*“After my husband’s death, living alone, you approach things with more anxiety because you don’t have the support of someone by your side, you feel empty…”.*

(I9)

However, family support can also lead to feelings of discomfort or guilt, particularly when patients feel they are imposing on their loved ones or have lost some independence:

*“… I feel worse for my children because I’m a bother to them.”*

(I9)

*“… I couldn’t come here alone, and that’s a discomfort because I always have to depend on someone, whereas before I was independent in everything…”*

(I26)

The dual role of family support highlights its significance as both a source of emotional stability and, at times, a cause of discomfort for patients. These findings emphasize the need for tailored interventions that address the complexities of interpersonal dynamics, ensuring that patients receive meaningful support without feelings of dependency or guilt.

3. Coping Strategies for the Wait

Patients described employing a variety of strategies to manage the emotional challenges of waiting for their diagnostic tests. These approaches reflect their efforts to maintain a sense of calm and avoid overthinking during a potentially stressful period. Many patients coped by diverting their attention from the diagnostic test to focus on daily activities, such as shopping or running errands, which provided a reassuring sense of normalcy:

*“… going shopping, running errands… that reassures me…”*

(I9)

*“… I try not to think about it until the last moment… I focus on work, home…”*

(I13)

Others reported using music as a distraction. Listening to portable headphones helped them remain calm and resist negative thoughts:

*“I try to distract myself, with music in my headphones. I mustn’t think about it, I have to stay calm; otherwise, if I start thinking, I’ll feel worse”*

(I2)

Social interaction also proved to be an effective coping mechanism for some patients. Conversations with others in the waiting room fostered a sense of mutual encouragement and provided comfort through shared experiences:

*“… I talk a lot with others, and it helps me relieve tension… encouraging others, but in encouraging others, I encourage myself, and time passes” *

(I7)

*“… hearing testimonies, the comparison with people who are surely worse off than I am, makes me feel better”*

(I27)

For many patients, spirituality and faith offered a powerful source of strength and hope. Trust in God and prayer helped them cope with uncertainty and gain a sense of peace in facing their situation:

*“… I’m a believer, I believe in the creator… faith is essential… prayer… that gives me a lot of strength…”*

(I7)

*“… well, if it is Christ’s wish, unfortunately, there’s nothing else I can do. I trust in faith, prayer, and common sense…”*

(I15)

*“… I have a lot of trust; I believe in God…”*

(I24)

The variety of coping strategies described by patients underscores the deeply personal nature of their responses to waiting. These approaches—from practical distractions to the comfort of faith and peer support—highlight the importance of addressing individual needs. Tailored interventions that leverage these strategies could further alleviate the emotional burden of waiting and enhance patient well-being.

4. Waiting Requires Attention to the Person and the Environment

The interviews underscored the importance of considering both the human and environmental aspects of the waiting experience to provide better support for patients undergoing diagnostic exams. Patients consistently highlighted the need for initiatives that enhance their sense of being welcomed and cared for while waiting.

Many expressed a desire for staff specifically trained to facilitate their reception, emphasizing the value of clear communication and assistance during check-in:

*“… those working here should be trained differently because training them and transferring different skills from what they currently have would certainly yield better results.”*

(I11)

*“… the presence of volunteers to help us understand the paths, how to move around… to provide support, give directions, take a number—this would certainly help a lot…”*

(I22)

*“… someone to let you know if there’s still more waiting…”*

(I24)

Another recurring need was for improved communication regarding exam results and interactions with radiology staff. Suggestions included activating remote communication services to provide updates and avoid the necessity of returning in person:

*“… communication when we’re at home, and the numbers and references when we’re home could be improved, because it’s not always possible to return in person to get an answer.”*

(I22)

Patients also identified psychological support as a valuable resource for alleviating anxiety and fear during the waiting period:

*“Maybe a psychologist would be useful… I know, in general, it can be a good approach; some people need it to cope with this anxiety, this fear.”*

(I3)

*“… psychological support… having some comfort would be important.”*

(I28)

The physical environment emerged as another significant focus. Patients highlighted the need for spaces that felt more comfortable, less chaotic, and better organized. Suggestions included adding screens to provide clear updates about wait times, background music, or television to reduce stress and improve the overall experience:

*“… having a more comfortable environment with less confusion… if there are other people like me who don’t know, they start asking questions and get agitated, and that agitation tends to spread to others.”*

(I22)

*“… I’m handling it pretty well, but it would be nicer if there were more space or a screen to show when it’s your turn.”*

(I12)

*“… background music would not be a bad idea, for those that are a little more anxious, it could be a distraction.”*

(I1)

*“Help them unwind, give them a chance not to think about what they’re about to do. For example, do you have TVs? Use them… background music.”*

(I2)

*“… a television, something to distract them so they don’t think so much… the television might help reduce anxiety a bit.”*

(I5)

*“Environmental comfort could be achieved by showing something on TV; it is a good distraction, and you stop thinking about what you have to do.”*

(I29)

*“… they could add something to make the environment more welcoming, more like home, soften the colors in the waiting area, making it feel more welcoming and less cold.”*

(I12)

This theme highlights the need to address both personal and environmental factors to improve the waiting experience for patients. By implementing staff training, psychological support, enhanced communication strategies, and environmental modifications, healthcare services can create a more patient-centered and less stressful waiting environment.

## 4. Discussion

The objective of this study was to explore the experiences lived by patients with CM before undergoing diagnostic examinations. The qualitative analysis of the interviews revealed a complex range of emotions and reactions that patients experience while waiting for a diagnostic exam. Individual, social, and environmental factors influence these experiences, generating emotional responses that can vary from anxiety and fear to optimism and calmness.

The results indicate that waiting for a diagnostic exam is a critical moment, often characterized by anxiety and worry. These emotions can stem from the fear of negative results or the uncertainty about preparing for the exam itself. The literature supports these observations, showing that uncertainty associated with the diagnosis can intensify anxiety in patients [26]. The idea that waiting can amplify the emotional experience is confirmed by studies demonstrating how the anticipation of stressful events can negatively impact psychological well-being [27]. This phenomenon is not exclusive to melanoma patients, as research on individuals awaiting cardiac interventions has shown that prolonged waiting times can exacerbate stress and psychological distress. Similarly, studies on patients undergoing cancer screenings reveal that uncertainty during the waiting period amplifies emotional strain. These comparisons highlight that waiting, regardless of the medical condition, is a significant contributor to emotional distress and requires targeted strategies to reduce its psychological impact [28]. Some patients described positive coping strategies, such as optimism, listening to music, or talking with other patients. These approaches can facilitate a more serene waiting experience. The literature suggests that adaptive coping strategies, such as socialization and optimism, are associated with less anxiety and a better quality of life in cancer patients [29]. Additionally, sharing experiences with other patients can create a sense of community and mutual support, contributing to a more positive perception of waiting [7].

Family support plays a crucial role in the lives of melanoma patients, with both positive and negative effects. On one hand, the support of loved ones provides comfort and reassurance, contributing to the patient’s emotional well-being. For example, one patient shared: “*This gives me peace of mind: seeing my children doing well, calm, and then I’m calm too*” (I8), while another stated, “*I’m happy like this, with my wife, my daughter, with my family and my daughter’s family… they’re doing well, and so are we”* (I20). These reflections highlight the emotional stability that family can provide during challenging times.

Many studies demonstrate that social support is fundamental in managing medical stress and illness [30], improving emotional adjustment and quality of life, and reducing the sense of dependence [31,32]. This support helps patients maintain a positive attitude and greater resilience in the face of disease challenges [31,32]. On the other hand, some patients perceive a dependence on family support, generating feelings of guilt and sorrow as they see their autonomy reduced and fear becoming a burden to their loved ones. As one patient noted, “*I feel worse for my children because I’m a bother to them*” (I9), while another remarked, “*I couldn’t come here alone, and that’s a discomfort because I always have to depend on someone, whereas before I was independent in everything*” (I26). These examples underline the emotional complexity of relying on family support, which can simultaneously comfort and distress patients. The literature confirms this: some studies report that patients experiencing chronic illness may feel guilty about the emotional burden their health places on their families [33].

Melanoma patients adopt various strategies to cope with the waiting period before a diagnostic exam, trying to manage anxiety and stress. Some patients prefer not to think about the exam until the last moment, focusing on daily activities such as grocery shopping or running errands to keep their minds occupied. The literature suggests that distraction can be an effective strategy for managing stress and anxiety [34]. However, evidence indicates that certain coping strategies may be more effective than others. For instance, distraction techniques like listening to music or engaging in daily tasks provide immediate relief by redirecting attention, but social interactions in the waiting room offer additional benefits by fostering a sense of community and mutual support. Studies have shown that social support not only reduces stress but also enhances emotional adjustment and resilience [35]. Similarly, spirituality and prayer provide deep internal reassurance for some patients, particularly those with strong personal beliefs, making these strategies highly effective in promoting long-term emotional stability. These findings highlight the importance of offering diverse coping options to accommodate individual preferences and needs.

Other patients use music as a means of distraction, listening to it through portable headphones to stay calm and avoid negative thoughts. Recent studies have shown that music can have beneficial effects on patients’ emotional and psychological states, reducing anxiety and improving mood [36]. Another common strategy is interacting with other patients in the waiting room. This exchange allows them to encourage each other and create a supportive environment. Sharing testimonials and experiences with those facing similar situations helps them feel better, often realizing that others may be in a more difficult condition than them. The literature highlights that social support and interaction with others in similar situations can improve emotional adjustment and reduce feelings of isolation [34]. Finally, some patients find comfort in prayer and faith. Spirituality plays an important role for many, offering a source of strength and tranquility. Trusting in God and praying becomes a way to cope with uncertainty and find hope in the process they are experiencing. The literature suggests that spirituality and religion may be correlated with a better quality of life [37].

Paying attention to the person undergoing the diagnostic exam is fundamental. Ensuring a warm and professional welcome can make patients feel respected and understood, improving their overall experience. A “warm and professional welcome” involves several key practices. Warmth is conveyed through empathetic behaviors, such as staff greeting patients with kindness, maintaining eye contact, and using a calm and reassuring tone. Professionalism is demonstrated through clear and efficient communication, providing timely updates about waiting times or procedures, and promptly addressing patient concerns. Furthermore, offering assistance to patients who may feel lost, such as having volunteers or staff available to guide them and answer questions, creates an environment of trust and support. These practices together help patients feel valued and reduce their emotional distress during the waiting period [38].

Requests for a more welcoming environment and better communication with healthcare staff have been strongly emphasized. Patients highlighted the need to improve communication and information regarding result retrieval and consultation with radiology staff. The ability to access remote communication and consultation services has been indicated as crucial in reducing anxiety and improving patient satisfaction. The literature suggests that a comfortable hospital environment and clear communication can reduce anxiety and improve the patient experience [38]. The implementation of improved welcome practices and clear information on healthcare processes has been associated with greater patient satisfaction [39]. Additionally, many patients emphasized the importance of psychological support to address anxiety and fear related to the diagnostic exam. Access to a psychologist could provide fundamental emotional support, helping patients better manage stress and anxiety. The literature highlights that psychological support can be crucial in improving the emotional well-being of cancer patients [40,41]. The waiting room environment is another factor that influences the patient experience. Preferences may vary significantly across demographics. Older patients might prioritize accessible seating and clear signage, while younger patients may prefer interactive distractions like digital screens or music. Cultural backgrounds can also shape preferences, with some valuing quiet spaces for reflection and others preferring vibrant designs. Gender differences might play a role too, as women may appreciate calming elements like soft colors or music, whereas men might focus more on functionality. Adapting waiting rooms to these diverse needs could enhance patient satisfaction and comfort [38,39].

A more comfortable environment, with less confusion, more space, and informative screens, can help reduce anxiety. Patients suggested the presence of screens indicating when it is their turn, background music, TVs, and more welcoming decor to create a less cold and more relaxing environment. The literature suggests that a comfortable and welcoming environment can improve patient experience and reduce stress [42]. Finally, music, TV, and decor can help distract patients during the wait, reducing anxiety and improving their emotional state. Studies have shown that distraction elements like music and television can have positive effects on patient well-being [43]. In summary, these interventions can help create a more serene and reassuring environment, helping patients better manage the waiting period and improving their overall experience.

## 5. Conclusions

The study revealed that waiting for a diagnostic exam is a critical moment for patients with cutaneous melanoma (CM), often characterized by anxiety and worry. Oncology healthcare professionals need to recognize these emotions and implement strategies to manage them. Promoting positive coping strategies, such as optimism and socialization, can improve the experiences of oncology patients. Additionally, balanced family support is crucial, as it can provide comfort but also create emotional dependence. Encouraging patients to keep their minds occupied with daily activities can help manage stress. Facilitating interaction among patients in the oncology waiting room can create a supportive environment, enhancing emotional adjustment. Recognizing the importance of spirituality can offer oncology patients strength and tranquility.

Investing in oncology healthcare staff training, psychological interventions, and technological innovation will significantly improve the waiting experience for cancer patients. Providing a warm and professional reception, along with clear and accessible communication, can reduce anxiety and improve patient satisfaction in oncology settings. Access to psychological support is crucial for addressing anxiety related to diagnostic exams. Creating comfortable waiting room environments with distraction elements, such as music and TV, can greatly enhance the oncology patient’s experience.

However, it is also essential to address the physical structure of the healthcare environment. Negative aspects such as inadequate furnishings and the lack of private rooms can exacerbate patient anxiety and discomfort. On the other hand, positive interventions at the personnel level, including training healthcare staff to be empathetic and responsive, can greatly enhance the overall patient experience.

Improving the functional and operational structure is equally important. Streamlining communication processes and reducing waiting times can significantly reduce patient stress and improve satisfaction. Efficient operational processes ensure that patients feel informed and respected throughout their healthcare journey.

These implications confirm the importance of a multidimensional approach in oncology clinical practice, as the quality of the waiting period can significantly influence the patient care pathway. Investing in these areas would not only foster a more welcoming and reassuring environment for oncology patients but also enhance their psychological well-being and overall satisfaction with oncology care pathways.

This study also revealed some aspects that can be useful indicators of quality of care, such as patient satisfaction, anxiety and stress levels, patient–provider communication, timeliness of care, psychological support services, physical environment quality, and staff training and empathy. By focusing on these quality indicators, future research can provide valuable insights into improving the overall patient experience and outcomes in oncology care settings.

### Limitations

The study presents several limitations, including monocentricity, which compromises the ability to generalize based on the results, given that it was conducted in a single location, Rome. This may influence patients’ experiences due to cultural and contextual factors. Additionally, the qualitative nature of the data can introduce subjectivity and difficulties in replicating the results, while researcher bias may further compromise interpretation. For the future, it is recommended to examine the influence of the disease stage on patients’ emotional experiences, exploring how progression impacts anxiety, coping mechanisms, and emotional needs. Moreover, it is recommended to conduct multicentric studies to obtain a larger sample, to adopt mixed methodologies for a more comprehensive view of patients’ experiences, and to integrate family support into intervention programs. It is essential to develop practical resources for managing anxiety and to train health professionals in recognizing and managing patients’ emotions. Finally, future research should continue to explore the emotional and relational dimensions of waiting, promoting a patient-centric approach in clinical practice.

## Figures and Tables

**Table 1:** Guiding questions for interviews.

Could you share with us how you are experiencing the waiting period before undergoing a diagnostic radiology investigation? ○ *If positive:* What factors contributed to this positive experience? What obstacles could have affected this experience? ○ *If negative:* What factors made this experience negative? What factors could have helped to improve the experience? Do you have any suggestions for improving the patient waiting period before a diagnostic radiology investigation?

**Table 2:** Example of the coding process in inductive analysis.

Codes	Categories	Themes
“I feel awful because the wait is long… there are many thoughts running through my mind…” (I18)	Negative Aspects	Waiting Affects the Emotional Experience
“… I’m still waiting for them to call me for the CT scan” (I22)		
“… the waiting time is long… the wait has always been devastating” (I18)		
“The waiting times… entering and finding out you have to wait another half hour is un-bearable. That half hour feels endless.” (I2)		
“…since it’s an ultrasound where I have to drink, it stresses me a little because I’m not sure if my bladder will be full or if I’ll be able to hold it.” (I17)	Anxiety About Preparation and results	
“… the wait creates anxiety… will I be able to do the bladder ultrasound?” (I23)		
“If something negative comes up, of course, it would lead to a spiral of worries.” (I6)		
“If there are significant changes in the report, I would be worried…” (I29)		
“… you don’t know if there is something wrong or not, and this creates a state of fear, a constant sense of suspense… there’s no one to tell you everything is fine.” (I24)		
“The fear of recurrence…” (I10)	Fear of Recurrence	
“… So I’ll have to remove the tumor, I’ll have to go through the whole process again, and so some fear comes in…” (I15)		
“… fear of what lies ahead, knowing that these melanomas can cause metastases…” (I19)		

**Table 3:** Sociodemographic and clinical data.

	*n*.	%
**Age (mean)**	57.4 anni	
**Gender**		
M	20	66.67
F	10	33.33
**Marital status**		
Married	22	73.33
Single	4	13.33
Divorced	1	3.33
Widow/widower	1	3.33
Data missing	2	6.67
**Education**		
Middle school	1	3.33
High school	12	40.00
Degree	17	56.67
**Employment**		
Employed	12	40.00
Retirees	12	40.00
Unemployed	1	3.33
Student	1	3.33
Data missing	4	13.33
**Previous oncological therapies**		
Yes	30	100.00
No	0	0.00
**Diagnostic test to be performed**		
CT scans	15	50.00
Ultrasound	10	33.33
MRIs	5	16.67

## Data Availability

The original contributions presented in this study are included in the article/Supplementary Material. Further inquiries can be directed to the corresponding author.

## Supplementary Materials

The following supporting information can be downloaded at: https://www.mdpi.com/article/10.3390/healthcare13080845/s1, File S1: COREQ checklist.
