# Supplementary material for: Exploring Cutaneous Melanoma Patients’ Experiences with Follow-Up Radiology: A Qualitative Study
Source: Healthcare (Basel). 2025 Apr 8;13(8):845. doi: 10.3390/healthcare13080845 (PMC12027287; doi:10.3390/healthcare13080845)
Supplement: Supplementary file 1 [file healthcare-13-00845-s001.zip › healthcare-3502329-supplementary.pdf]

Supplementary File S1: COREQ checklist

Consolidated criteria for reporting qualitative studies (COREQ): 32-item checklist

Tong A, Sainsbury P, Craig J. Consolidated criteria for reporting qualitative research (COREQ): a 32-item checklist for interviews and focus groups. International Journal for Quality in Health Care. 2007. Volume 19, Number 6: pp. 349 – 357

Item No	Guide Questions/Description	Reported on Page #
Domain 1: Research team and reflexivity		
Personal Characteristics		
1. Interviewer/ facilitator	Which author/s conducted the interview?	Two researchers carried out the interviews (FG, SM).
2. Credentials	What were the researcher's credentials? E.g., PhD, MD	FG has a PhD. SM is a PhD student.
3. Occupation	What was their occupation at the time of the study?	FG and SM were both nurses.
4. Gender	Was the researcher male or female?	Females.
5. Experience and training	What experience or training did the researcher have?	Both had received training in qualitative research methodologies and had previous experience using this methodology.
Relationship with participants		
6. Relationship established	Was a relationship established prior to study commencement?	No prior relationship was established between the researchers and participants.
7. Participant knowledge of the interviewer	What did the participants know about the researcher? e.g., personal goals, reasons for doing the research?	Participants knew where the researchers worked and the purpose of the research.
8. Interviewer characteristics	What characteristics were reported about the interviewer/facilitator? e.g., Bias, assumptions, reasons and interests in the research topic	SM was a nurse in an oncology setting and was interested in the subject of study. FG had a strong interest in the subject of interest.
Domain 2: study design		
Theoretical framework		
9. Methodological orientation and Theory	What methodological orientation was stated to underpin the study? e.g., grounded theory, discourse analysis, ethnography, phenomenology, content analysis	A qualitative descriptive, inductive and monocentric approach was chosen.

Item No	Guide Questions/Description	Reported on Page #
		The Framework Analysis approach was used for data analysis.
Participant selection		
10. Sampling	How were participants selected? e.g., purposive, convenience, consecutive, snowball	A purposive sample of patients with cutaneous melanoma had been recruited.
11. Method of approach	How were participants approached? e.g., face-to-face, telephone, mail, email	Interviste semi-strutturate, faccia a faccia, guidate da domande chiave
12. Sample size	How many participants were in the study?	A total of thirty patients were recruited.
13. Non-participation Setting	How many people refused to participate or dropped out? Reasons?	Only 1 patient did not agree to participate in the study because he was engaged in activities related to his professional work.
14. Setting of data collection	Where were the data collected? e.g., home, clinic, workplace	The interviews took place in the hospital, in a dedicated room, ensuring an uninterrupted environment and ensuring comfort and privacy for the participants.
15. Presence of nonparticipants	Was anyone else present besides the participants and researchers?	No. Only interviewers and patient interviewees were present.
16. Description of sample	What are the important characteristics of the sample? e.g., demographic data, date	Participants completed a demographic data form (age, gender, education level, marital status, occupation, family structure, number of children) and clinical information (disease, previous oncological therapies, current treatment, type and reason for the investigation).
Data collection		
17. Interview guide	Were questions, prompts, and guides provided by the authors? Was it pilot tested?	The semi-structured questions were developed by the research team based on information obtained from a literature review, were submitted to expert judgement and then tested on 6 patients.
18. Repeat interviews	Were repeat interviews carried out? If yes, how many?	No.

Item No	Guide Questions/Description	Reported on Page #
19. Audio/visual recording	Did the research use audio or visual recording to collect the data?	Interviews were audio-recorded.
20. Field notes	Were field notes made during and/or after the interview or focus group?	No.
21. Duration	What was the duration of the interviews or focus group?	The average length of the interviews was 24 minutes.
22. Data saturation	Was data saturation discussed?	Data saturation was briefly described in the "Sample and setup" section.
23. Transcripts returned	Were transcripts returned to participants for comment and/or correction?	Transcripts were not returned to participants.
Domain 3: analysis and findings		
Data analysis		
24. Number of data coders	How many data coders coded the data?	Data were coded and categorized independently by three researchers, while other researchers verified the consistency of these codes, categories, subthemes, and themes.
25. Description of the coding tree	Did the authors provide a description of the coding tree?	An example of the coding process in the inductive analysis was provided in Table 2 and the "Data Analysis" section.
26. Derivation of themes	Were themes identified in advance or derived from the data?	Themes were derived from the data.
27. Software	What software, if applicable, was used to manage the data?	NVivo
28. Participant checking	Did participants provide feedback on the findings?	No.
Reporting		
29. Quotations presented	Were participant quotations presented to illustrate the themes/findings? Was each quotation identified? e.g., participant number	Quotations have been presented in the results section, with participant codes assigned to all participants and used against quotations.
30. Data and findings consistent	Was there consistency between the data presented and the findings?	We endeavored to report the study findings in a clear, consistent manner to accurately reflect the data that have been collected.

Item No	Guide Questions/Description	Reported on Page #
31. Clarity of major themes	Were major themes clearly presented in the findings?	Yes, major themes are clearly presented in the results section.
32. Clarity of minor themes	Is there a description of diverse cases or a discussion of minor themes?	We committed to discussing all the data that emerged from the interviews.
